# Is theology more of a field than a father is a king? Modelling semantic relatedness in processing literal and metaphorical statements

**DOI:** 10.3758/s13423-022-02072-6

**Published:** 2022-03-22

**Authors:** Chris Westbury, Parastoo Harati

**Affiliations:** Department of Psychology, University of Alberta, P220 Biological Sciences Building, Edmonton, AB T6G 2E9 Canada

**Keywords:** Word meaning, Text comprehension, Semantics, Reading

## Abstract

One major question in the study of metaphors historically is: Are different mechanisms involved in the comprehension of figurative statements versus literal statements? Many studies have addressed this question from a variety of perspectives, with mixed results. Following Harati, Westbury, and Kiaee (*Behavior Research Methods*, 53, 2214-2225, 2021), we use a computational (word embedding) model of semantics to approach the question in a way that allows for the quantification of the semantic relationship between the two keywords in literal and metaphorical “x is a y” statements. We first demonstrate that almost all literal statements (95.2% of 582 statements we considered) have very high relatedness values. We then show that literality decisions are slower for literal statements with low relatedness and metaphorical statements with high relatedness. We find a similar but smaller effect attributable to the cosine of the vectors representing the two keywords. The fact that the same measurable characteristics allow us to predict which metaphors or literal sentences will have the slowest literality decision times suggests that the same processes underlie the comprehension of both literal and metaphorical statements.

## Introduction

Despite their ubiquity in language, cognitive science still has a long way to go in providing a model of metaphor comprehension mechanisms. The significance of these linguistic units lies in their ubiquity, complex structure, and abstractness. Their abstractness is of the utmost cognitive importance (e.g., Inhelder & Piaget, 1964), as cognitive research has shown that people’s perception of the world is altered by the adoption of an abstract mindset (Gilead et al., 2014). The complex nature of metaphors allows for the impact of many different factors on their comprehension, creating many questions.

Writing in 1725, the anti-Cartesian philosopher Giambattista de Vico (1725) noted that “metaphor makes up the great body of the language among all nations” (p. 104). In more recent times, many researchers have supported de Vico’s claim that metaphors are not just common in ordinary language use but fundamental to it. In his book discussing figurative language, Gibbs (1994) answered his question “Why should poetic imagination matter to cognitive science?” by criticizing the alternate idea that is sometimes expressed:

An old but prevailing idea among students of mind holds that thought and language are inherently literal. […] the traditional view of mind is mistaken, because human cognition is fundamentally shaped by various poetic or figurative processes. Metaphor, metonymy, irony, and other tropes are not linguistic distortions of literal mental thought, but constitute basic schemes by which people conceptualize their experience and the external world. (p. 1; for similar arguments, see, e.g., Fauconnier & Turner, 2002; Geary, 2011; Hofstadter & Sander, 2013; Lakoff & Johnson, 1980).

One of the major factors affecting the study of metaphors historically has been the ways in which metaphor processing differs from literal statement processing. An ongoing question in the field has been: Are there different mechanisms involved in the comprehension of figurative statements versus literal statements? As we outline below, most major views of metaphor comprehension propose that metaphoric and literal statements are processed similarly. However, systematic identification of quantifiable differences that account for observed differences in processing literal and metaphorical statements is a gap that has not been addressed. In this paper we use computational modelling to address this gap.

Two traditional major theoretical approaches to metaphor comprehension are *the indirect access approach* and *the direct access approach*.

*The indirect access approach* proposed that non-literal meanings are accessed only after the literal meaning has been rejected (Janus & Bever, 1985). The observed differences in time to accept a statement as either literal or figurative were explained by the necessity of this additional step for figurative meanings. This approach considers no impact of context for lexical access. Supporters of this approach to metaphor processing in pragmatics argue that metaphors present a scenario in which Grice’s (1989) maxim of quality is violated. This view also assumes a replacement of an initial reading of the literal meaning with a reading of the figurative meaning. The study by Clark and Lucy (1975) was one of the first influential studies that presented support for the three-stage model, in which understanding a sentence “arises from a recipe requiring three ingredients: (1) the literal meaning of the sentence, (2) the perceived context, and (3) a so-called conversational postulate” (p. 57). The need for re-considering a non-literal statement results in longer reading times for indirect statements like metaphors than for direct statements.

More recent work has supported *the direct access approach* (e.g., Gibbs, 1994, 2002; Sperber & Wilson, 1986; Vu et al., 1998), which posits that processing of metaphor and literal statement follow similar paths (e.g., Blasko & Connine, 1993; Glucksberg et al., 1982; Keysar, 1989; McElree, Gerring & Healy, 1983; McElree & Nordlie, 1999). Unlike indirect access, this approach predicts that context can affect lexical access, rejecting the assumption of a need for automatic literal meaning access. According to theories based on this approach, context prepares the figurative meaning in advance during lexical access, which allows for it to be already available at the interpretation stage.

Many theories emerged providing empirical evidence in support of either of these approaches, each focusing on a different aspect of metaphors. Ortony et al. (1978) argued that Clark and Lucy’s (1975) design affected their results, as they presented their sentence items without the context that might make them easier (faster) to understand. Ortony et al. (1978) proposed a view in which comprehension is seen to occur in the interaction between the statement and the context, suggesting that literality or metaphoricity is not a determiner of the comprehension process. This view is sometimes referred to as *the interactionist view*. Their work is an example of the initial research that suggested acquiring literal meaning is not always faster than figurative meaning.

A view that provides support for the direct access approach is *the categorization view* (Glucksberg et al., 1997; Keysar, 1989), which assumes that metaphors are comprehended as categorical assertions and involve dual reference, meaning that the source^1^ of a metaphor signifies both a basic-level concept and also a superordinate conceptual category which contains the target. According to this view, metaphors, just like literal statements, are processed directly without having to reject the literal meaning first for the figurative processing to start. Under this view, literal comprehension has no inherent advantage over metaphorical comprehension.

A competing view to the categorization view is *the comparison view,* which assumes that metaphor comprehension requires a comparison of the basic meaning of the metaphor with the meaning that its context evokes. One of the most referenced models under this view is the structure-mapping model (Gentner, 1983; Gentner & Bowdle, 2008). According to this model, a metaphor is a mapping between the domains of the target and source. This alignment occurs with a structural alignment stage (the juxtaposition of the target and the source) and a projection stage (projection of inferences of the target to the source), respectively. Evidence in support of the comparison view has shown that the presence of context decreases the speed of processing metaphorical statements to almost the same speed as processing their literal counterparts (Gibbs & Gerrig, 1989; Ortony et al., 1978).

Giora (1997, 1999, 2003) proposed a model called *the graded salience hypothesis*. According to Giora, the only difference between literal and figurative interpretation is in their attributed properties. These properties are activated by the source. This hypothesis posits that two mechanisms of bottom-up (lexical information) and top-down (contextual information) "run in parallel" to generate the appropriate meaning (Giora et al., 2015, p.2). The speed of processing is a function of matching the interpretation with meaning that is activated either by the word or the context. The distinction between literality and metaphoricity is not in the nature of the processing but in the properties that are activated based on frequency, familiarity, and conventionality.

Some researchers argue that the answer to identifying the difference in processing is quantitative rather than qualitative. In other words, processing metaphors is more difficult and requires more effort, but the cognitive processes underlying their comprehension are the same. Instances of research arguing in support of this view come from neuroscience studies. For example, in an event-related potential (ERP) study, Coulson and Van Petten (2002) suggest that the difficulty in metaphor processing is due to the higher semantic distance between the domains to which the target and source belong (compared to literal statements), while the cognitive processes involved in literal and metaphoric comprehension are essentially the same. As we also propose here, this view suggests that while there may be a difference in processing effort, the reason for the extra effort is not due to any differences in the cognitive functions brought to bear on the problem.

In another ERP study, Weiland et al. (2014) also state that a cost induced by the computation of the relation between the target and source is responsible for their observed Late Positivity, which is “sensitive to semantic distance between source and target” (p. 14).

Metaphor type is also a major factor influencing metaphor processing (Cardillo et al., 2010; Cavazzana & Bolognesi, 2020; Werkmann et al., 2021). Because of their ease of generation, transparent target-source relationship, and structural homogeneity, the most common type of metaphor studied in psychology research is the nominal (“x is a y”) metaphor, which involves nouns as target and source. In order to facilitate the comparison of our results to previous findings, we focus on such metaphors here.

Computational models of metaphor processing have based their models on the foundation of some of these views, and can therefore help with adjudicating the claims made by these theories. For example, Kintsch (2000, 2008) and Utsumi and Sakamoto (2007) drew partly upon the categorization view. Perhaps the most prominent computational model of metaphor processing is Kintsch’s (2000) predication algorithm, which has provided the foundation for many other computational models. Kintsch’s model of metaphor processing conceptualizes the categorization view in terms of spreading activation through a network of semantically related words. The spreading activation in a nominal metaphor was simulated using a vector model of language (LSA; Landauer & Dumais, 1997**)**, in three steps:

i) The nearest *m* cosine neighbors of the source word (y) are identified.
ii) From among those *m* words, the *k* neighbors that have vectors with the highest cosine similarity to the vector of the target word (x) and the source word (y) are identified.
iii) The *k* vectors are averaged together with the target vector to make a new vector.

The cosine distance between this new vector and the vector of the source word was proposed as a measure of metaphor comprehensibility. In essence, Kintsch’s model works by nudging the target vector into alignment with the dominant meaning of the source vector and then quantifying how close that alignment has brought the two vectors.

Harati et al. (2021) extended Kintsch’s model by systematically assessing the parameters *m* and *k* in the model, and by assessing the effect of averaging the *k* vectors with the vectors of either or both of the source and target words. Kintsch has posited values of between 500 and 100 for *m* and 5 for *k*. Harati et al. (2021) demonstrated that the optimal values for predicting human judgments of metaphor quality were *k* = 5 and *m* = 4,500, with the common vectors averaged into both the source-word and the target-word vectors. They called the cosine distance between the two vectors 5-5-4500, because it was one of many *k1-k2-m* parameter sets they considered. To make its interpretation transparent in the present context, we refer to the distance they called *5-5-4500* as *Relatedness*. High values of Relatedness suggest that the aligned meanings of the source and target words are very close, while low values of Relatedness suggest that those aligned meanings are not close.

In the current study, we test the hypothesis that the processing advantage for literal statements versus novel nominal metaphors reflects the fact that literal statements usually have higher degrees of relatedness between their adjusted x and y vectors and higher Cos values between their unadjusted x and y vectors.

## Method

### Participants

Participants were 66 university students (52 (78.8%0 female), with an average (SD) age of 21.8 (6.9) years. They participated in return for partial course credit. Fifty (75.8%) were undergraduate students. Seven (10.6%) had a bachelor’s degree. One (1.5%) had a graduate degree. Sixty (90.9%) described themselves as being right-handed. Although our experiment description had specified that participants needed to be right-handed, three participants (4.5%) described themselves as ambidextrous and three (4.5%) described themselves as being left-handed. All participants attested to being native English speakers, defined as having learned to speak English before the age of 4 years.

### Stimuli

**Fig. 1:**
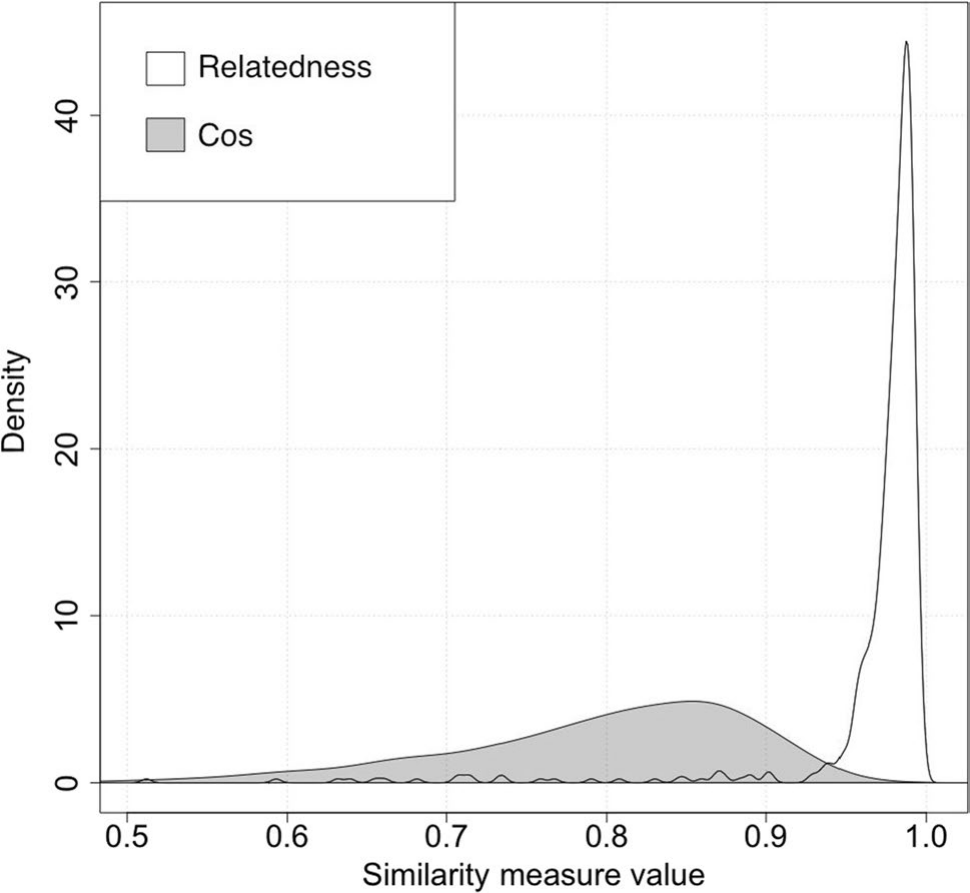
Density plots of the distribution of Relatedness and Cos values across 582 literally true sentences

Our goal in creating the stimuli was to find literally true statements that showed a range of Relatedness and Cos values. It is relevant to the claims of this paper that this was difficult to do, because most literally true “x is a y” statements have very high Relatedness values (Fig. 1). After several iterations of effort to create literally-true “x is a y” statements with words that were not high on Relatedness values, we ended up with 582 literally true statements in total. Of those, 95.2% had Relatedness values > 0.9. However, there were a few with lower values. Examples of literally true statements with relatively low Relatedness values include “A mood is a state”^2^ (0.56), “A collection is a group” (0.66), and “A president is a human” (0.71). Examples of literally true statements with very high Relatedness values include “Wheat is a grain” (0.997), “A photograph is a picture” (0.997), and “A cat is a feline” (0.996). We selected the 40 sentences with the lowest Relatedness values (reproduced in Appendix 1). In selecting these stimuli, we did not concern ourselves with Cos because, as shown in Fig. 1, this value is relatively widely distributed across literally true statements.

**Fig. 2:**
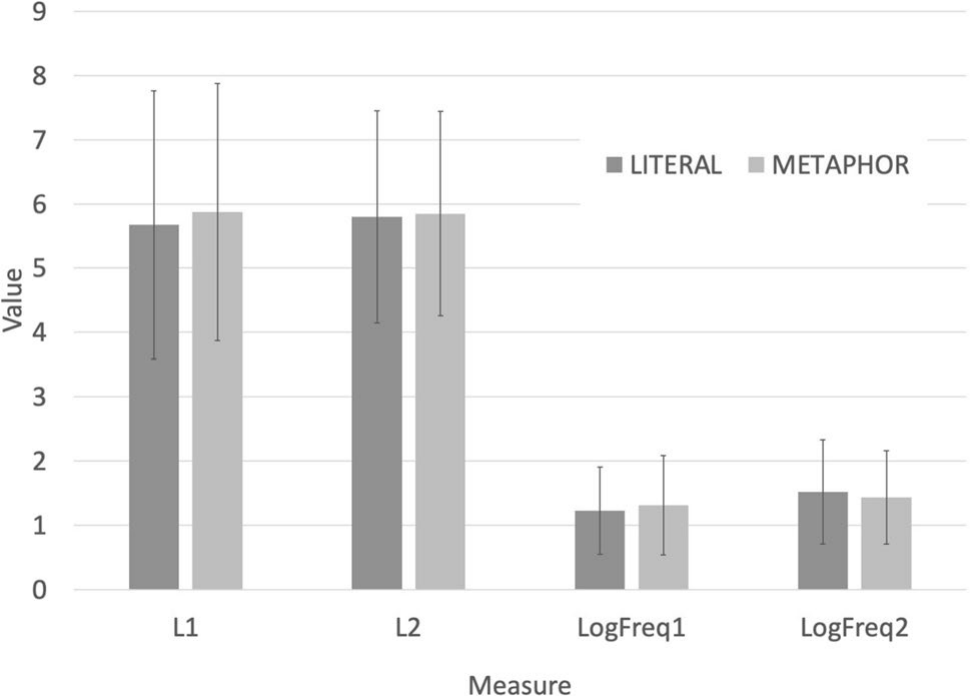
Match on length and logged frequency (LogFreq) between metaphors and literal statements for the first and second key words (x and y) in literally true and metaphorical “x is a y” statements. Bars are SD

We matched each of these stimuli to a metaphor on the normalized length and normalized logged frequency of the two keywords, x and y. To do this, we used all 622 metaphors from Harati et al. (2021). We first found the closest match between the two keywords x and y from all 40 literal statements and the keywords from all the metaphors. We then removed that pair and repeated the exhaustive search for the most closely matched remaining pair, until all 40 literal sentences had been matched. This algorithm guarantees a very close match (Fig. 2). The average (SD) summed difference was 0.21 (0.084), or an average per-measure difference of 0.21/4 = 0.053z per measure. The worst-matched pair had a summed difference over the four normalized measures of 0.37, a per-measure difference of 0.37/4 = 0.09z.

### Procedure

The experiment was conducted online on the experimental platform testable.org, after being ethically reviewed and accepted by the University of Alberta Research Ethics Board. After reading a description of the experiment and their rights, participants gave informed consent to participate by clicking on a button. They were then shown the following instructions:

“In this experiment, we want you to decide whether or not sentences are literally true. A true sentence is a sentence like ‘The earth is a planet’, which states a true fact. Half the sentences we will show you are literally true. The other half are metaphors. If a sentence is literally true, please hit the 'c' key [for 'correct']. If it is not literally true, please hit the 'x' key [for 'incorrect']. Please use the first and second fingers of your dominant hand to hit the keys, as we get a better measurement of your reaction time when you use one hand. Please make your decision as quickly as you can, without sacrificing accuracy.”

Stimuli were presented on the middle of the screen in 36-pt Times font, with an intertrial interval (ITI) of 800 ms. Sentences remained on the screen until a response was obtained. Every participant made decisions about all 80 sentences.

The data are available at https://osf.io/ke6yh/.

## Results

Before further analysis, we removed all responses shorter than 400 ms (122 responses, or 2.3% of all responses) or longer than 5,000 ms (134 responses, or 2.5% of all responses) on the grounds that such responses were unlikely to reflect attentive responding. From the remaining responses, we eliminated responses that were more than 3 SDs (= 3 * 838.6 ms) from the mean response time (RT) (1,591.3 ms). This eliminated an additional 134 responses (2.5% of all responses) for being too slow.

For the key RT analysis, we eliminated the incorrect responses, leaving 4,181 data points. We analyzed those data with linear mixed-effects modelling, using the lme4 package (Bates et al., 2015) running in R 3.6.0.

The model development is summarized in Tables 1 and 2. Importantly for the focus of this study, Cos entered in interaction with sentence type (*t* = 6.26, *p* = 4.38e-10) and so did Relatedness (*t* = 6.88, *p* = 7.08e-12).

**Table 1:** Summary of model development for predicting correct literality decision response times (RTs)

	Name	Model	AIC	AIC Diff	R^2^	R^2^ Diff	Accepted?
Random effects	M1	(1 + ID)	65840	[BASE]	0.4596	[BASE]	Yes
	M2	M1 + (1 | ORDER)	65710	-130	0.5202	0.0606	Yes
Fixed effects	M3	M2 + Length1	65691	-19	0.5230	0.0028	Yes
	M4	M3 + Length2	65647	-44	0.5300	0.0070	Yes
	M5	M2 + Length1 * Length2	65636	-11	0.5314	0.0014	Yes
	M6	M5 + LogFreq1	65629	-7	N/A	N/A	Did not enter
	M7	M5 + LogFreq2	65629	-7	N/A	N/A	Did not enter
	M8	M5 + Cos * Relatedness * SentenceType	65477	-159	0.5496	0.0182	Yes

**Table 2:** Model for predicting correct literality decision response times (RTs). Continuous predictors have been normalized. Default sentence type is metaphor

Predictor	Estimate	SE	df	t	p
(Intercept)	1659.74	56.46	188.48	29.4	<2E-16
Relatedness	-317.93	58.97	4052.95	-5.39	7.38E-08
SentenceType * Cos	-274.99	43.96	4045.46	-6.26	4.38E-10
Cos	205.69	34.21	4046.07	6.01	1.99E-09
Length2	56.99	9.3	4051.41	6.13	9.83E-10
SentenceType [Literal/Metaphor]	-55.4	37.11	4057.26	-1.49	0.14
Length1	30.61	10.13	4052.2	3.02	0.0025
Length1 * Length2	30.82	11.38	4056.75	2.71	0.0068
Cos * Relatedness	-414.01	60.21	4051.50	-6.88	7.08E-12
SentenceType * Relatedness	341.59	59.79	4053.13	5.71	1.19E-08
SentenceType * Cos * Relatedness	372.7	62.28	4051.43	5.98	2.36E-09

**Fig. 3:**
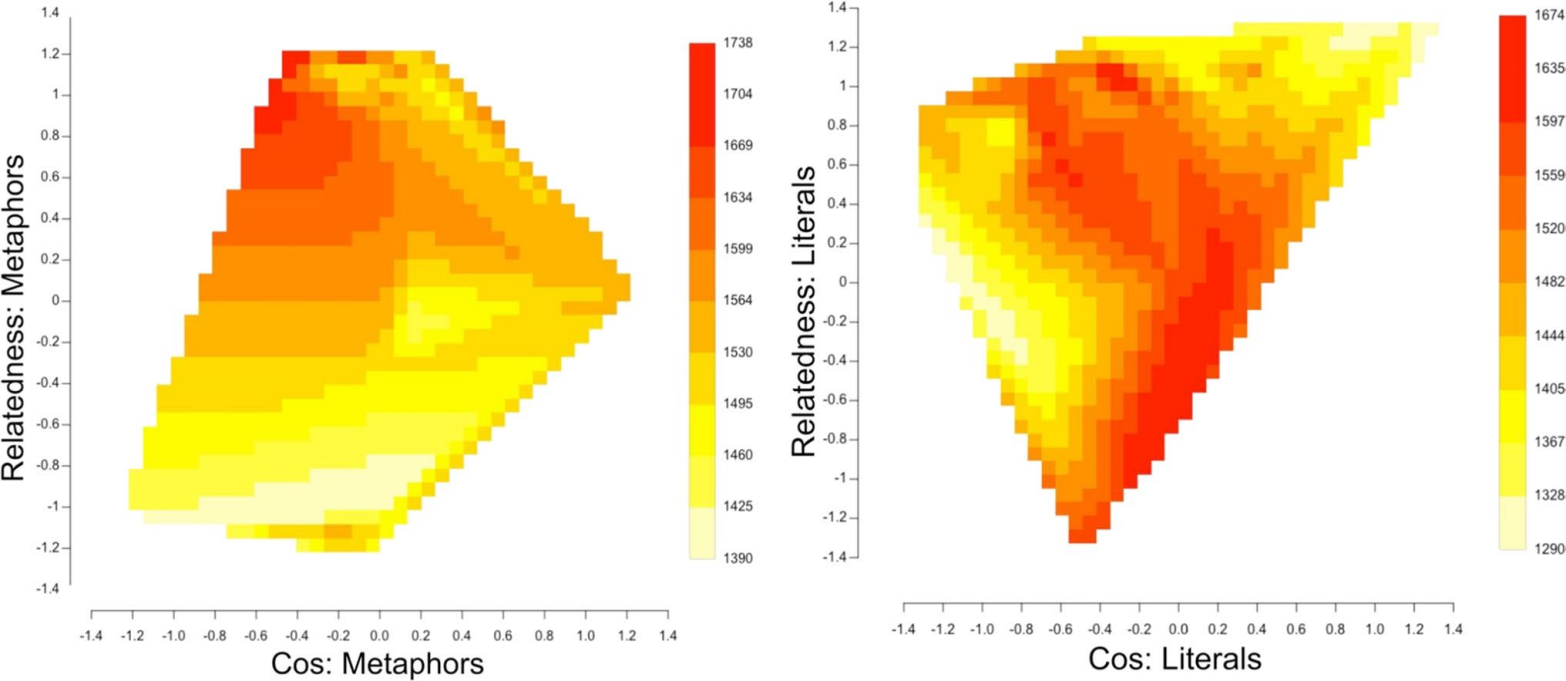
Three-way interaction for correct-decision response times (RTs) between Cos, Relatedness, and sentence type

The three-way interaction was also statistically significant (*t* = 5.98, *p* = 2.36e-09). Adding the three-way interaction reduced the AIC by 159, indicating a substantial reduction in the likelihood of information loss in the more complex model that included that interaction. It improved the R^2^ of the estimated to the observed values by 1.8%, which is more than the improvement (1.12%) from adding the two words lengths in interaction to the base model that included only random effects of participant and stimulus order. To better understand this interaction, we divided up the data into metaphors and literal statements, and constructed LME models on each of these subsets. Both models included Cos, Relatedness, and their interactions. Their relation is illustrated in Fig. 3 (created using R-package contourPlot; Murphy, 2020). For metaphors, a low Cos (suggesting the two words are generally not closely related semantically) coupled with a high relatedness value (suggesting that the two words *are* closely related semantically along the particular dimension captured by their common neighbours) is associated with slower RTs. Examples of metaphors with this property are “Desire is an animal” (zCos = -1.29; zRelatedness = 0.46) and “A poet is a locksmith” (zCos = -1.06; zRelatedness = 0.69). For literal statements, sentences with words that have a high Cos and a high Relatedness have the quickest RTs. These are sentences such as “A rifle is a gun” (zCos = 1.88; zRelatedness = 0.84) or “A snake is reptile” (zCos = 2.04; zRelatedness = 0.85). However, so do sentences containing word pairs with a low Cos, across the midrange of Relatedness values. These are sentences such as “Africa is a place” (zCos = -1.81 zRelatedness = -0.60), “Theology is a field” (zCos = -1.33; zRelatedness = -1.16), and “A cat is a being” (zCos = -1.18, zRelatedness = 0.15). We speculate that decisions are made rapidly to these sentences because the only way to have a low Cos in a literally true sentences is to make the sentence extremely general, as exemplified by these examples.

**Fig. 4:**
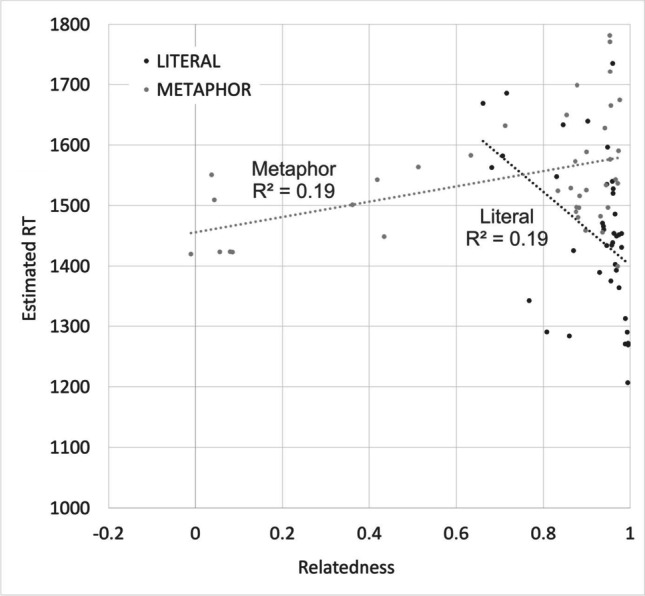
Estimated decision times for 40 “x is a y” metaphors and 40 literal “x is a y” statements matched on word frequencies and lengths (as shown in Fig. 2), as a function of Relatedness

The relationship between Relatedness and RT by sentence type is shown in Fig. 4. Estimated literal sentence decision RTs take longer when the two words have lower Relatedness (*r* = -0.441). Metaphors show the opposite effect, with decisions being made more quickly when the two words have lower relatedness (*r* = 0.436).

**Fig. 5:**
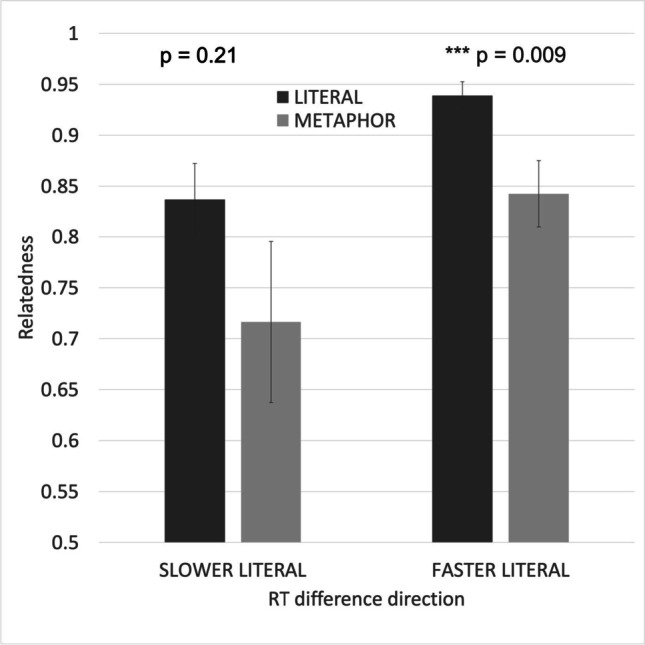
Difference in Relatedness for matched literal/metaphor sentence pairs in which the literal statement was correctly classified more slowly (**left**) or more quickly (**right**) than the metaphor

Correct decisions to literally true statements with low Relatedness are made more slowly than correct decisions to many metaphors. Among the 40 matched pairs of literal and metaphorical sentences, correct decisions were made more slowly for the literal than the metaphorical sentence in nine pairs. The differences in Relatedness among these nine pairs are contrasted to the Relatedness difference among the other 31 pairs in Fig. 5. There is no reliable difference in Relatedness among the nine pairs that showed a metaphor advantage (*p* = 0.21 two-tailed). In contrast, Relatedness is significantly lower for metaphors than for literal statements among the 31 pairs that showed a literality advantage (*p* = 0.009).

**Fig. 6:**
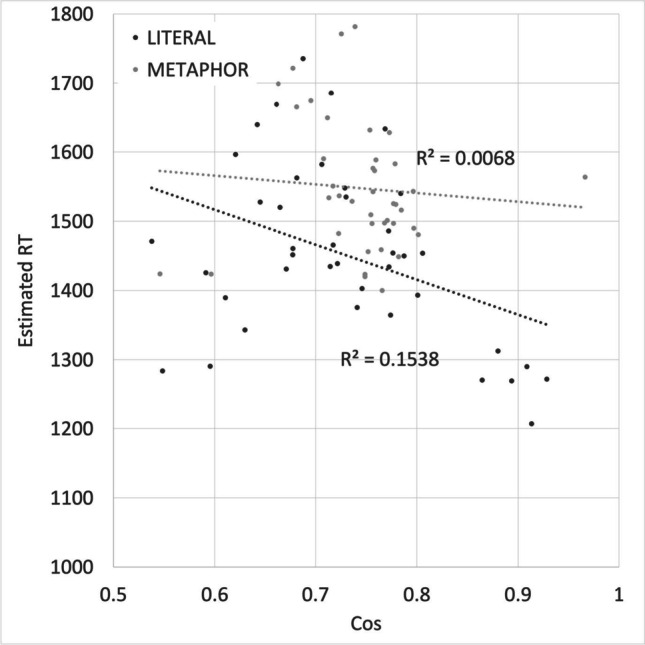
Reaction times for 40 “x is a y” metaphors and 40 literal “x is a y” statements matched on word frequencies and lengths (as shown in Fig. 2), as a function of Cos

Figure 6 shows the relationship between Cos and correct decision times for metaphors and literal statements. Literal sentence decisions are faster when the two words have a lower Cos (*r* = -0.392). There is no effect of Cos on decision time for metaphors (*r* = -0.080). There was no reliable difference in Cos between the nine sentence pairs that showed a metaphor advantage (*p* = 0.07 two-tailed) or between the 31 pairs that showed a literality advantage (*p* = 0.95).

**Fig. 7:**
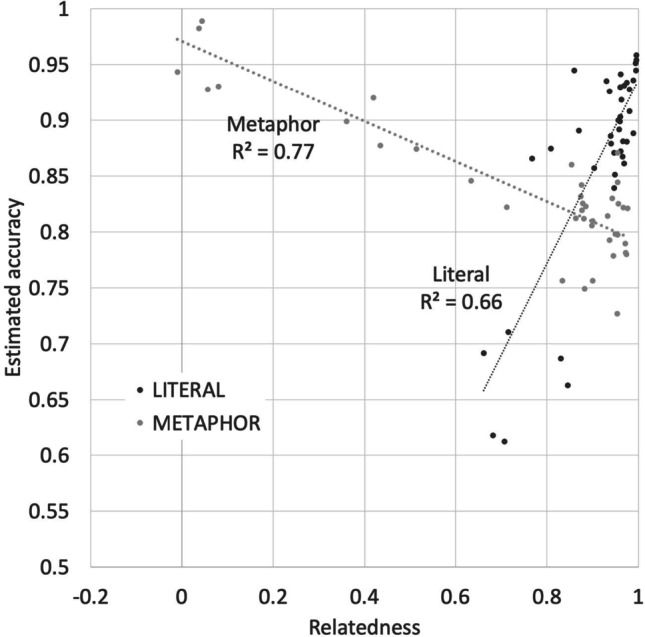
Estimated accuracy for 40 “x is a y” metaphors and 40 literal “x is a y” statements matched on word frequencies and lengths (as shown in Fig. 2), as a function of Relatedness

**Fig. 8:**
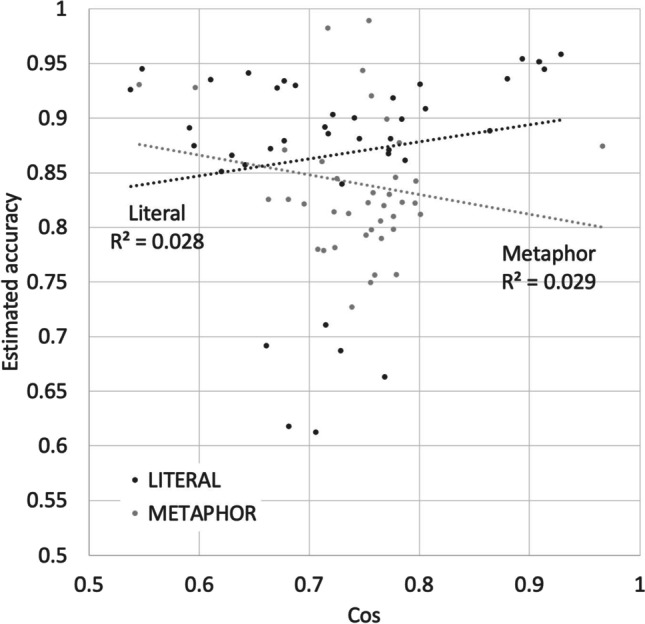
Estimated accuracy for 40 “x is a y” metaphors and 40 literal “x is a y” statements matched on word frequencies and lengths (as shown in Fig. 2), as a function of Cos

The accuracy results mirror these decision time results. The model development is summarized in Table 3, with the best model shown in Table 4. As with the decision time results, there was a significant three-way interaction between Cos, Relatedness, and Sentence Type. The effect of Relatedness is graphed in Fig. 7. Participants were more accurate at classifying the literal sentences they responded to most quickly, those with high relatedness. They were less accurate at classifying the metaphors they responded to most slowly, also those with high relatedness. Figures 7 and 8 shows that the results were similar in direction but attenuated in strength for the role of Cos.

**Table 3:** Summary of model development for predicting literality decision accuracy

	Name	Model	AIC	AIC Diff	Accepted?
Random effects	M1	(1 + ID)	3278	[BASE]	Yes
	M2	M1 + (1 | ORDER)	3280	2	No
Fixed effects	M3	M1 + Length1	3224	-56	Yes
	M4	M3 + Length2	3225	1	No
	M5	M1 + Length1 * Length2	3232	8	No
	M6	M3 + LogFreq1	3199	-25	Yes
	M7	M3 + LogFreq2	3208	9	No
	M8	M1 + Length1 * LogFreq1	3196	-3	No
	M9	M6 + Cos * Relatedness * SentenceType	3076	-123	Yes

**Table 4:** Model for predicting literality accuracy. Continuous predictors have been normalized. Default sentence type is metaphor

Predictor	Estimate	SE	df	t	p
(Intercept)	0.718	0.0214	226.2	33.54	<2E-16
Relatedness	0.290	0.0267	4816	10.85	<2E-16
SentenceType [Literal/Metaphor]	0.109	0.0170	4816	6.42	1.50E-10
Cos	-0.107	0.0159	4816	-6.69	2.48E-11
Length1	-0.034	0.00573	4816	-6.00	2.15E-09
LogFreq1	-0.023	0.00532	4818	-4.33	1.53E-05
Relatedness * SentenceType	-0.335	0.0273	4816	-12.30	<2E-16
Cos * Relatedness * SentenceType	-0.179	0.0290	4816	-6.19	6.48E-10
Cos * Relatedness	0.163	0.0280	4816	5.81	6.72E-09
Cos * SentenceType	0.088	0.0209	4816	4.18	2.98E-05

## Discussion

This study investigated whether the literal versus metaphoric language processing difference could be explained through computational modelling. Our results suggest that the RT advantage sometimes seen for recognizing literal sentences over figurative sentences may in part be an effect of the semantic distance between the words in the sentences. Literally true sentences are generally processed more quickly than metaphors because they generally have more closely related words in them. When we decrease the strength of the relation between the words in a literal sentence, decisions may take longer than they do for metaphors of the same form with words matched on frequency and length. The literality advantage may also reflect the nature of the literality decision task. In literality decision tasks such as the one we used, it is a safe bet that if the words in a sentence are very closely related, it is a not metaphor.

Our findings argue against Glucksberg’s assumption of dual-reference, which proposes a categorical distinction between literal and figurative representations of the source in the lexicon (see also Roncero, 2013). In our model there is a continuity of relatedness from a high-relatedness literal statement like “A shark is a fish” (Relatedness = 0.985) through a lower-relatedness literal statement like “A shark is a predator” (Relatedness = 0.977) to a lower-relatedness non-literal statement like “A shark is a lawyer” (Relatedness = 0.961), and on from there to even lower relatedness nonsense statements like “A shark is an eggcup” (Relatedness = 0.874).

Our findings are roughly consistent with assumptions of the graded salience hypothesis. Giora (2003) and Roncero (2013) argue that it is the activation of all salient properties of the words in a sentence that determines whether the final interpretation is figurative or literal. Roncero (2013) wrote that:

“First, a set of salient associated properties is initially activated when a vehicle is a read within a metaphor or simile, but the structure has the effect of making connotative properties more salient for metaphors, and denotative properties more salient for similes. These different activation levels for the activated properties can then cause metaphors to evoke interpretations that seem more figurative. The advantage of this framework is the elimination of issues related to whether meaning is initially literal or figurative, or whether context alone is sufficient for determining the correct interpretation. Instead, both the words within a sentence and the context itself are predicted to affect the salient properties that are activated. People then deduce from that set of salient properties the appropriate interpretation for a given sentence.” (p. 207)

In our model, the context-relevant salient properties of a particular sentence are captured by the overlap between the neighbours of the two words.

Both Kintsch’s predication algorithm (2000) and the categorization view of metaphor processing focus on nominal metaphors. The resemblance of the syntactic form of “x is a y” metaphors to that of literal sentences accelerates the speed of processing metaphorical statements (Glucksberg & Keysar, 1990). Our claims are thus far limited to novel, simple nominal metaphors. Within that domain, the evidence presented lends further support to the view considered in Gibbs and Gerrig (1989) that “Identical mental processes drive the comprehension of both literal and metaphorical utterances” (p. 148), by identifying well-defined characteristics that allow us to predict which metaphors and literal sentences will have the slowest literality decision times.

## Appendix 1: Stimuli used in the literality decision experiment

**Table:**

Sentence	Type	COS	Relatedness	Length1	Length2	LogFreq1	LogFreq2
A jam is a spread.	LITERAL	0.715	0.715	3	6	0.80	1.55
Love is a feeling.	LITERAL	0.772	0.965	4	7	2.43	1.65
Alcohol is a drug.	LITERAL	0.864	0.989	7	4	1.27	1.89
Theology is a field.	LITERAL	0.596	0.808	8	5	1.04	2.19
A sidecar is an attachment.	LITERAL	0.688	0.960	7	10	-0.64	1.21
Baseball is a business.	LITERAL	0.661	0.661	8	8	1.13	2.67
A glove is a covering.	LITERAL	0.645	0.961	5	8	0.42	1.36
Painting is a process.	LITERAL	0.642	0.903	8	7	0.81	2.35
Skin is a tissue.	LITERAL	0.880	0.989	4	6	1.40	0.84
A bat is a mammal.	LITERAL	0.801	0.968	3	6	1.13	0.11
A bowl is an object.	LITERAL	0.630	0.767	4	6	1.51	1.78
Butter is a food.	LITERAL	0.715	0.958	6	4	1.01	2.08
A rifle is a gun.	LITERAL	0.913	0.994	5	3	0.98	1.75
Science is a process.	LITERAL	0.620	0.948	7	7	2.22	2.35
A son is a male.	LITERAL	0.671	0.980	3	4	2.12	1.66
A ring is an object.	LITERAL	0.722	0.960	4	6	1.50	1.78
A gun is a weapon.	LITERAL	0.894	0.995	3	6	1.75	1.42
A cat is a being.	LITERAL	0.611	0.930	3	5	1.81	2.79
A planet is a sphere.	LITERAL	0.787	0.969	6	6	1.77	0.80
A butterfly is a flier.	LITERAL	0.730	0.947	9	5	0.41	-0.35
An integer is a number.	LITERAL	0.538	0.937	7	6	0.93	2.45
Nausea is a sickness.	LITERAL	0.784	0.959	6	8	0.11	0.61
A hide is a skin.	LITERAL	0.772	0.946	4	4	1.57	1.40
America is a land.	LITERAL	0.729	0.830	7	4	2.29	2.02
A seed is a nut.	LITERAL	0.741	0.956	4	3	1.16	0.99
Africa is a place.	LITERAL	0.548	0.859	6	5	1.50	2.40
A whip is a weapon.	LITERAL	0.776	0.963	4	6	0.83	1.42
A product is an object.	LITERAL	0.769	0.845	7	6	2.20	1.78
A snake is a reptile.	LITERAL	0.928	0.995	5	7	0.89	-0.20
A wake is a gathering.	LITERAL	0.717	0.939	4	9	1.31	1.33
A frog is an amphibian.	LITERAL	0.909	0.993	4	9	1.35	-0.62
A hammer is a tool.	LITERAL	0.746	0.966	6	4	1.26	1.67
Biochemistry is a field.	LITERAL	0.681	0.681	12	5	0.31	2.19
A bible is a text.	LITERAL	0.774	0.974	5	4	1.99	1.97
Wickedness is a vice.	LITERAL	0.706	0.706	10	4	0.28	1.58
Wine is a liquid.	LITERAL	0.677	0.974	4	6	1.29	1.03
A kitchen is a place.	LITERAL	0.591	0.869	7	5	1.13	2.40
Granite is a mineral.	LITERAL	0.806	0.980	7	7	0.19	0.54
A bill is a charge.	LITERAL	0.678	0.940	4	6	2.20	1.79
Gravity is a force.	LITERAL	0.665	0.961	7	5	1.26	2.10
An emotion is a danger.	METAPHOR	0.76	0.95	7	6	0.82	1.46
A day is a struggle.	METAPHOR	0.77	0.88	3	8	2.61	1.34
Language is a tool.	METAPHOR	0.76	0.88	8	4	2.01	1.67
Laughter is an engine.	METAPHOR	0.75	0.71	8	6	0.80	1.66
Dullness is a nightmare.	METAPHOR	0.73	0.95	8	9	-0.80	0.98
Education is a business.	METAPHOR	0.74	0.95	9	8	2.05	2.67
Greed is a illness.	METAPHOR	0.71	0.85	5	7	0.96	1.20
Anxiety is a teacher.	METAPHOR	0.97	0.51	7	7	0.93	1.64
Youth is an arrow.	METAPHOR	0.75	0.04	5	5	1.25	0.67
A wife is a crutch.	METAPHOR	0.80	0.88	4	6	1.92	0.13
A myth is a highway.	METAPHOR	0.55	0.08	4	7	1.19	1.49
Lust is a fire.	METAPHOR	0.75	0.09	4	4	0.70	1.98
Beauty is a joy.	METAPHOR	0.77	0.97	6	3	1.20	1.27
Science is a creator.	METAPHOR	0.76	0.90	7	7	2.22	1.33
The past is a guide.	METAPHOR	0.76	0.90	4	5	2.18	1.54
A pen is a machine.	METAPHOR	0.80	0.97	3	7	1.43	1.87
A day is a battle.	METAPHOR	0.78	0.95	3	6	2.61	1.56
A girl is a light.	METAPHOR	0.60	0.06	4	5	1.83	2.23
Desire is an animal.	METAPHOR	0.66	0.88	6	6	1.68	1.63
Prejudice is a noose.	METAPHOR	0.71	0.94	9	5	0.84	0.14
A garden is a church.	METAPHOR	0.68	0.96	6	6	1.20	2.21
Kinship is a treasure.	METAPHOR	0.77	0.94	7	8	-0.21	0.78
A child is a gift.	METAPHOR	0.75	0.94	5	4	2.05	1.40
A father is a king.	METAPHOR	0.72	0.97	6	4	1.94	1.91
Racism is a rat.	METAPHOR	0.72	0.93	6	3	1.23	0.99
A grave is a truth.	METAPHOR	0.78	0.88	5	5	1.20	2.28
A smile is a candle.	METAPHOR	0.76	0.87	5	6	1.16	1.33
A story is a window.	METAPHOR	0.78	0.63	5	6	2.16	1.79
Pride is a manhole.	METAPHOR	0.76	0.42	5	7	1.20	-0.61
Chaos is an architect.	METAPHOR	0.72	0.04	5	9	1.09	1.43
A poet is a locksmith.	METAPHOR	0.68	0.95	4	9	0.84	-0.50
A belief is a door.	METAPHOR	0.77	0.36	6	4	1.72	1.78
Truthfulness is a force.	METAPHOR	0.78	0.83	12	5	-0.12	2.10
Power is a risk.	METAPHOR	0.78	0.43	5	4	2.42	1.95
Parenthood is a fire.	METAPHOR	0.75	-0.01	10	4	0.28	1.98
Fame is a killer.	METAPHOR	0.80	0.88	4	6	0.99	1.14
Harmony is a power.	METAPHOR	0.74	0.86	7	5	0.89	2.42
A dreamer is a thinker.	METAPHOR	0.70	0.98	7	7	0.27	0.71
Hope is a battle.	METAPHOR	0.78	0.90	4	6	2.20	1.56
A patient is a child.	METAPHOR	0.71	0.97	7	5	1.41	2.05

## Footnotes

1 In this study we use the terms *target* and *source* interchangeably with *topic* and *vehicle*, respectively. The *target/topic* refers to what is being discussed, and the *source/vehicle* refers to the concept used to discuss the target.

2 Note that one effect of Kintsch’s model is to disambiguate the intended meaning of ambiguous words like *state*, since the adjusted vector of the word *state* is adjusted towards its shared meaning with *mood.*
